# An Interesting Case of Acute Asymptomatic Lead Perforation of a Permanent Cardiac Pacemaker

**DOI:** 10.7759/cureus.13334

**Published:** 2021-02-14

**Authors:** Anunay Gupta, Sourabh Agstam, Tushar Agarwal, Sunil Verma

**Affiliations:** 1 Cardiology, Vardhman Mahavir Medical College and Safdarjung Hospital, New Delhi, IND; 2 Cardiology, All India Institute of Medical Sciences, New Delhi, IND

**Keywords:** pacemaker lead perforation, pacemaker lead displacement, impending pericardial effusion, pacemaker complication

## Abstract

Acute complications of pacemaker implantation such as lead dislodgement, pneumothorax, and myocardial perforation are not uncommon. Management of these usually requires reintervention. We herein describe lead perforation after a single chamber pacemaker implantation, which was successfully managed conservatively. This case underscores that vigilant monitoring post lead perforation can avoid a redo procedure.

## Introduction

Acute complications such as lead dislodgement, pneumothorax, and myocardial perforation are not uncommon after pacemaker implantation. Lead perforation can be either early or late, and lead can perforate through the myocardium, into the epicardial space, pericardium, or chest wall [1]. Such perforations can sometimes be clinically occult and not accompanied by symptoms such as pain or pericardial effusion [2]. A chest X-ray in two different views is useful in demonstrating perforation but is limited by its inability to differentiate between the ventricular cavity, myocardium, and pericardium. A cardiac computed tomography (CT) is more reliable for lead tip identification. Such a case is usually managed by repositioning the leads at the desired position, at the risk of pericardial effusion, infection, and prolonged admission. We herein present a case of an 80-year-old gentleman who was managed conservatively following lead tip perforation into the left ventricular apex.

## Case presentation

**Figure 1:**
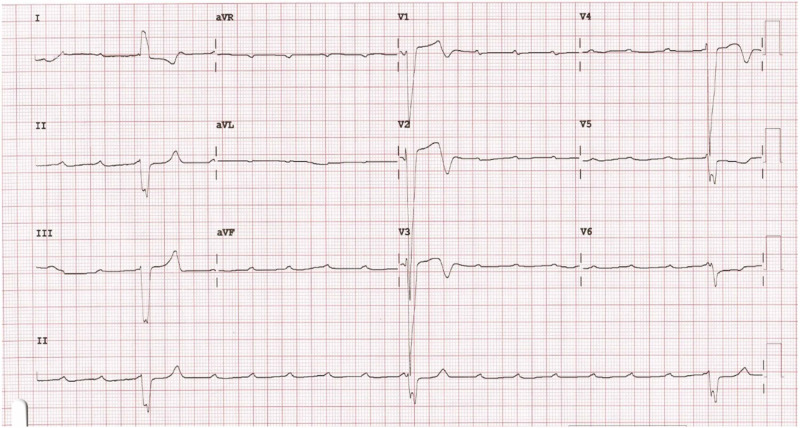
12-lead-electrocardigram (ECG) showing complete atrio-ventricular dissociation with baseline heart rate of 15/minute, suggestive of complete heart block

An 80-year-old male presented in the emergency department with symptoms of recurrent syncope for one day. He was a chronic smoker with a smoking index of 200 pack years without any other comorbidities. At presentation, the pulse rate was 15/minute, blood pressure was 90/60 mmHg, and respiratory rate was 14/minute. The 12-lead electrocardiogram (ECG) showed complete atrioventricular dissociation with a heart rate of 15/minute, suggestive of complete heart block (CHB) (Figure 1).

**Figure 2:**
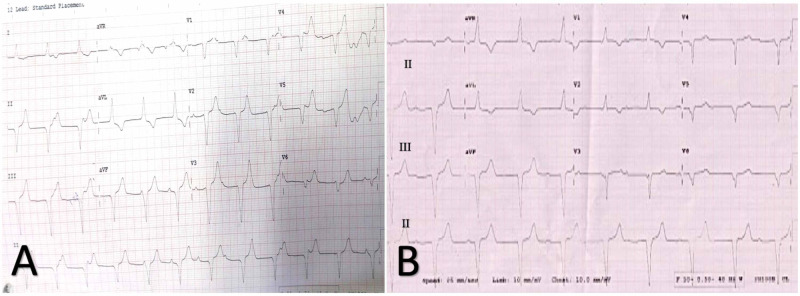
(A) 12-lead ECG showing left bundle branch block with left axis, suggestive of right ventricle apical pacing; (B) 12-lead ECG showing right bundle branch block with superior axis, suggestive of left ventricle apical pacing

Cardiac biomarkers were negative and after proper consent, he was taken up for single chamber permanent pacemaker implantation (VVIR) Sensia SESR01 Implantable Pulse Generator (Medtronic, Minneapolis, MN, USA) for symptomatic CHB due to financial constraints on part of the patient. The passive fixation lead (tined lead) was used at the right ventricular apex for ease of positioning and atraumatic implantation. The permanent pacemaker implantation procedure using a bipolar lead was uneventful with the optimal achievement of lead parameters and lead position fluoroscopically. Postoperative EKG showed a left bundle branch block with a heart rate of 60/minute, suggestive of right ventricular apical pacing (Figure 2A). The next day, ECG revealed a right bundle branch block heart rate of 60/minute, which was suggestive of left ventricular apical pacing (Figure 2B).

**Figure 3:**
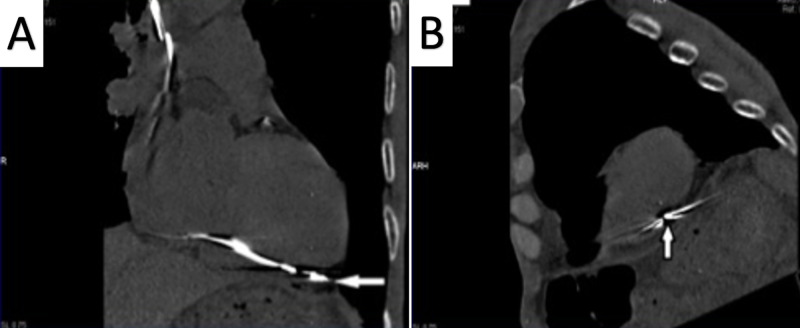
Non-contrast computed tomography of chest (oblique sagittal views) showing the lead tip (arrow) outside the right ventricular cavity and lying epicardially at the left ventricular apex in the pericardium

This raised the doubt of lead migration. Echocardiography revealed minimal pericardial effusion. CT scan showed the lead tip traversing intramyocardially through the right ventricular apex and lying near the left ventricular apex in the pericardial cavity (Figures 3A-3B).

Pacemaker interrogation revealed normal lead parameters in bipolar mode with R wave of 9.2 mV, Impedance of 680 ohms and threshold of 0.9 V at 0.4ms pulse width. There was no evidence of phrenic nerve capture.** **At this stage, there were two options either to reposition the intramyocardially impacted bipolar lead (with the risk of cardiac tamponade and subsequent consequences like infection and prolonged admission) or to manage the patient conservatively with close monitoring of lead position and parameters. After discussion with the patient, we opted for the second option and were able to discharge the patient after 10 days of monitoring and mobilizing him completely. After 12 months of follow-up, the patient is fine with excellent lead parameters and no pericardial effusion.

## Discussion

Symptomatic pacemaker lead perforation in the right atrium or right ventricle is uncommon, with an incidence of 0.1%-0.8% [1,2]. However, asymptomatic lead perforation as detected by CT scan occurs in 15% of patients, with a higher incidence in atrial leads as compared to ventricular leads [3]. Symptomatic perforations can present with chest pain, pneumothorax, hemopneumothorax, pneumopericardium, pericardial effusion, cardiac tamponade, and death [4-6]. The patient can present with vague chest pain radiating to the neck and shoulder, mistaken as musculoskeletal rather than pericarditic pain leading to delayed diagnosis [7,8]. Pacing parameters like capture threshold and sensing threshold may change depending on the new position of the lead tip. Syncope, heart failure, and cardiac arrest can occur following failure to pace. 

The causes of lead perforation are multifactorial. The pacemaker lead-related factors are excessive loop or tension on the passive fixation lead; an excessive number of turns to deploy the full helix and rotating faster than 1/second are risk factors in active fixation lead. Atrial leads, implantable cardioverter defibrillator leads, leads with a small diameter or a small tip surface-as well as the excessive length of the electrode are other risk factors. Patient factors include the thinner, dilated cardiac chambers, recent episode of myocardial infarction, temporary leads, steroid use, low body mass index (<20kg/m²), older age, female gender, and concomitant anticoagulation therapy [9].

A chest X-ray in the posterior-anterior and lateral view helps identify the position of lead but it cannot differentiate between the ventricular cavity, myocardium, and pericardium. Transthoracic echocardiography is an easily available non-invasive modality and can be diagnostic [10,11]. CT scan is currently the gold standard in the diagnosis of lead perforation [12,13]. The star artifact, related to the imaging of the metal implant, surrounding the electrode tip sometimes makes it difficult to precisely identify the lead tip. Magnetic resonance imaging (MRI) is not recommended for detecting lead perforation due to concerns about catastrophic complications, especially in older devices. However, with new generations of MRI-conditional devices, this imaging modality, with fewer lead artifacts compared with CT, may become the gold standard for the detection of lead perforation in the future. Currently, it is only performed with safety protocols in patients with other definite indications for MRI [14].

Surgical removal of the perforating leads is usually considered as the preferred strategy [5,15]. Atrial lead perforation usually requires open drainage. Percutaneous transvenous extraction with surgical backup is also a feasible option [6,16]. Acute lead perforation, which occurs during or shortly after implantation (<24 hours), maybe hemodynamically unstable and thus requires consideration for emergency treatment [6]. One study has reported altered lead parameters in 16 cases out of 18 patients with lead perforation and extracted all the leads [17]. Patients with asymptomatic lead perforations also show altered lead electrical parameters, necessitating removal and repositioning [17]. The extraction of a perforated lead in the asymptomatic patient even with abnormal lead parameters is not mandatory when the risks of repositioning outweighed the potential benefits in high-risk patients with comorbidities [18]. Lead parameters may or may not be significantly different between perforated and non-perforated leads [3,17]. Similarly, there was no significant change in lead parameters and impedance in our case, and should not be used as a parameter to rule out lead perforation. The plausible reason being that the lead anode is still in contact with the endocardium and the cathode is in contact with the epicardium [19]. Therefore, all lead perforations are not the same. There is considerable heterogeneity in the pacing parameters after an asymptomatic lead displacement and the approaches in managing them [11].

One study compared the complication rates in 22 patients managed conservatively versus in 26 patients managed by lead revision strategies for lead perforations [20]. Recurrent perforation-related symptoms, lead dysfunction, and device infection were similar in the two groups during 12 months of follow up. Nonetheless, cardiac tamponade developed in six of the conservative cohort against one in the lead revision cohort. Importantly though, five of the six in the conservative cohort who developed tamponade were treated by antiplatelets or anticoagulants during or shortly after the procedure [20]. This reiterates that careful patient selection is essential before a conservative approach is chosen. Therefore, a pacemaker leads in the pericardial cavity with more than acceptable pericardial effusion, altering pacing parameters, and in a patient requiring long term anti-platelets or anticoagulants necessitates repositioning. Recurrent pericarditic pain, lead dysfunction, and device infection were not more frequent with a conservative approach [20].

The main question confronting the authors was whether an acute lead perforation necessitated a repositioning; therefore, increasing the risk of infection, tamponade, and prolonged hospitalization in a frail 80-year old patient, especially when he was asymptomatic with no indication for antiplatelets or anticoagulation and normal lead parameters. The authors decided to manage the patient conservatively with close monitoring of lead position and parameters after discussions with the patient and relatives. Eventually, he was discharged after 10 days. This prevented a repeat intervention, and even after 12 months of follow-up, the patient remained asymptomatic with excellent lead parameters and no pericardial effusion. Such initial conservative management with close monitoring has been described previously when the risks of repositioning were felt to outweigh potential benefits in selected patients and is supported by this case report.

## Conclusions

To summarize, conservative management with watchful monitoring of asymptomatic acute lead perforation of a permanent pacemaker is a feasible option in patients with good lead parameters without significant effusion.
